# Strategies to Enhance the Efficacy of T-Cell Therapy for Central Nervous System Tumors

**DOI:** 10.3389/fimmu.2020.599253

**Published:** 2020-11-12

**Authors:** Deepak Upreti, David Bakhshinyan, Darin Bloemberg, Parvez Vora, Chitra Venugopal, Sheila K. Singh

**Affiliations:** ^1^McMaster Stem Cell and Cancer Research Institute, McMaster University, Hamilton, ON, Canada; ^2^Department of Surgery, Faculty of Health Sciences, McMaster University, Hamilton, ON, Canada; ^3^Department of Biochemistry and Biomedical Sciences, Faculty of Health Sciences, McMaster University, Hamilton, ON, Canada

**Keywords:** central nervous system tumors, CART, immune system, glioblastoma, T cells therapy

## Abstract

Mortality rates in patients diagnosed with central nervous system (CNS) tumors, originating in the brain or spinal cord, continue to remain high despite the advances in multimodal treatment regimens, including surgery, radiation, and chemotherapy. Recent success of adoptive cell transfer immunotherapy treatments using chimeric antigen receptor (CAR) engineered T cells against in chemotherapy resistant CD19 expressing B-cell lymphomas, has provided the foundation for investigating efficacy of CAR T immunotherapies in the context of brain tumor. Although significant efforts have been made in developing and translating the novel CAR T therapies for CNS tumors, including glioblastoma (GBM), researchers are yet to achieve a similar level of success as with liquid malignancies. In this review, we discuss strategies and considerations essential for developing robust preclinical models for the translation of T cell-based therapies for CNS tumors. Some of the key considerations include route of delivery, increasing persistence of T cells in tumor environment, remodeling of myeloid environment, establishing the window of treatment opportunity, harnessing endogenous immune system, designing multiple antigen targeting T cells, and rational combination of immunotherapy with the current standard of care. Although this review focuses primarily on CAR T therapies for GBM, similar strategies, and considerations are applicable to all CNS tumors in general.

## The Immune System and CNS Tumors

The Central nervous system (CNS) consists of the brain and spinal cord and coordinates most functions of the body and mind. Both the brain and spinal cord are protected by layers called the meninges and insulated by cerebrospinal fluid (CSF) (1–3). Additionally, the CNS is protected from infiltrating dangers, including toxins, pathogens, and circulating immune cells by a physical blood-brain barrier (BBB) (2). The BBB is restrictive in nature due to the tight junctions maintained by endothelial cells, which allow nutrients and small molecules to pass to brain tissues but restrict unwanted danger signals and pathogens (4).

Primary malignant CNS tumors are a substantial cause of death in both children and adults. The most prevalent brain tumors in children comprise of embryonal tumors, including medulloblastoma and atypical teratoid/rhabdoid tumor (ATRT) and Gliomas, that include ependymoma, pilocytic astrocytomas, and diffuse intrinsic pontine gliomas (5). On the other hand, the most frequent adult brain tumors are categorized into Meningiomas and Gliomas such as ependymomas, astrocytoma, oligodendrogliomas, and glioblastoma (GBM). Early diagnosis of tumors is important as localized tumors are manageable, whereas patients with malignant or disseminated tumors die of their disease (6).

Both genetic and environmental factors such as mutations in genes regulating DNA repair, cell cycle, inflammation, metabolism, and exposure to radiation were shown to be risk factors associated with CNS tumors (7, 8). In addition, the immune system in the brain differs from the rest of the body, as it lacks the functional adaptive immunity required for mounting a response against CNS tumors (8). The baseline immune system in the CNS is facilitated by microglia. Microglial cells are the resident macrophages of the CNS system, thus play an important role in scavenging damaged neurons, synapses, and pathogenic harmful stimuli. These cells also interact with neurons and modulate synapse formation, neuronal proliferation, differentiation, and migration (9, 10). These cells arise from myeloid precursors born in the yolk sac and invade during early embryonic development (9, 11, 12). Despite this, the CNS allows the circulation of peripheral immune cells *via* meningeal spaces. Separation of brain parenchyma from a continuous supply of peripheral immune cells is critical to maintaining the homeostasis of the organ (13). Microglia are present in the CNS during the early days of embryonic development and maintain the number of neural progenitors through phagocytosis, responding to tissue damage (14–16). Due to the influence of the brain environment, microglia are unique at the molecular level compared to tissue-resident macrophages and blood-derived macrophage (17–22). Adaptive immunity is invoked during chronic infection, autoimmunity, or cancer (23–25). T cells and T cell derived cytokines IL-4, IL-17, IFN-γ are implicated in cognitive function, as well as social dysfunction (26–28). Prior dogma stated that the CNS lacks an immune system, and only microglia participated in such interactions. But recent research shows that the meningeal lymphatics play important role and presence of adaptive immunity in CNS (29).

## Failure of Endogenous T Cells To Recognize CNS Tumors

Traditionally, it was thought that the CNS is an immune-restricted site. A number of factors, such as absence of histological lymphatics, existence of BBB, absence of adaptive immunity, rare presence of antigen-presenting cells, and downmodulation of MHC molecules in neuronal and glial tissue, contribute to endogenous T cell suppression in CNS tumors (8, 30–34). However, more recent data suggests that the CNS is under constant immunosurveillance (35). The CNS is surrounded by functional lymphatic vessels, providing gateways for immune cells into and out of the CNS (36). In addition to the rare presence of T cells in CNS tumors, it is likely that aggressive tumor growth of a tumor such as GBM, is also due to high ratio of suppressive myeloid cells to effector T cells, and this may be the major contributing factor to rapid growth of tumor and treatment resistance to immunotherapy (37, 38). Goswami et al. recently showed a high ratio of immunosuppressive myeloid cells compared to T cells in GBM. GBM has a higher abundance of CD68+ myeloid cells and CD73^high^ myeloid cells and these myeloid cells persisted after anti-PD1 therapy and correlate with reduced overall survival. Checkpoint therapy mediates protection against GBM when CD73 is deleted in mice, suggesting an immunosuppressive role for myeloid cells (38). Myeloid cells exert their immunosuppressive functions by secreting either soluble factors, or by direct cell-cell contact. Tumor-associated Macrophages (TAMs) secrete immunosuppressive cytokines TGF-B, IL-6, IL-10 that result in downregulation of costimulatory molecules and MHC expression lead to reduced phagocytic activity and reduced anti-tumor immunity. Moreover, TAMs also express cell surface receptors such as FAS ligand leading to apoptosis of T cells expressing FAS receptor (39, 40). T-cell senescence was reported in CNS malignant tumors with a CD4+CD28-CD57+ phenotype, which was correlated with lower survival of patients (41). Expression of exhaustive markers such as PD-1, CTLA-4, TIM-3, TIGIT, CD39 was also shown to contribute to T cell exhaustion in CNS tumors (42, 43). Other immunomodulatory cells and molecules such as MDSCs, Tregs and STAT3 and IDO respectively, were also involved in T-cell dysfunction (44–46). Overall, CNS tumors elicit T-cell dysfunction by inducing senescence, exhaustion, and apoptosis (47, 48).

Several tumors associated antigens are being targeted by CART or TCR based T cells therapy against CNS tumors in both preclinical and clinical settings. It must be noted that efficacy of a CAR T cell therapy in a PDX animal model does not guarantee translation of findings to humans in a clinical trial setting (49–53). Several factors such as route of administration, immunosuppressive tumor microenvironment, abundant presence of myeloid cells, role of endogenous immune system, timing of treatment may limit the therapeutic benefit of T cell therapies in humans with CNS tumors. Here, we highlight the enhancement of T cell-based therapies for CNS tumors.

## Factors to Consider For Designing T*-*Cell Therapies Against CNS Tumors

### Suitable Preclinical Models for Evaluating T-Cell-Based Therapy in CNS Tumors

Immunocompetent, patient-derived orthotopic and humanized mouse models are currently being used to evaluate the efficacy of T cell-based immunotherapy for CNS tumors. Immunocompetent mouse models such as, SMA-560, 4C8, GL261, GL26, and CT-2A offer advantages because tumor cells can be implanted into fully immune-competent mice and therefore recapitulate many features of the immune system, tumors, and stromal cell crosstalk. Syngeneic mouse models are inexpensive and easy to use (54), and T cell-based therapy evaluated in the presence of a functional immune system could provide a close resemblance to actual patients. Previously, it was shown that the administration of EGFRvIII mCAR-modified T cell therapy cured tumor-bearing mice with established intracerebral glioma (54). However, the efficacy was dependent on lymphodepletion, suggesting the benefit of utilizing syngeneic tumor models. Moreover, therapy- induced long-term protection, indicated the important role of the host endogenous immune system. Major limitations of syngeneic mouse models include the fact that immortal cell lines are implanted, antigens expressed are not always clinically relevant and T cell-based therapy requires further modifications before taking them to clinic. Furthermore, the modifications required for this transition are highly relevant and include: changing and/or humanizing binders and other CAR structural/signaling elements, adapting human-centric CAR-T generation and cellular manufacturing conditions, and accounting for inherent patient-related impacts on source material, notwithstanding additionally having to confirm that these modifications have not affected therapeutic efficacy.

In xenograft models, innate and adaptive immune systems of mice are eliminated, and human T cell-based therapy is given to treat human tumors. Earlier studies have shown that myeloid cells such as macrophages/monocytes are the major mediators of cytokine release syndrome associated with CART cell therapy (55). Major limitations of xenograft models are failure to predict toxicity from lack of myeloid-derived immune cells, inability to assess the impact of lymphodepletion, and inability to study the influence of endogenous immunity.

To study the toxicity associated with T cell-based therapy, influence of lymphodepletion and the role of other immune cells in humanized mouse models is being considered (56). Patient-derived primary tumor cells from human brain tumor tissue can be applied to CART cell studies to test efficacy. Recently, we tested the toxicity of CD133+CART cells by infusing CD133+CART cells in humanized CD34+ mice, and we observed no adverse effects on normal CD133+ cells (57). Humanized models are expensive for routine use in many laboratories with limited funding. However, these models recapitulate the best clinical scenario for T cells-based therapy, providing proof-of-principle studies of human CART cells, readily translatable to the clinic (**Table 1**).

**Table 1:** Advantages and limitations of several models which could be adopted for studying T-cell therapy for CNS tumors.

Model	Advantages	Limitations
Syngeneic	Presence of full immune system, stromal cells, inexpensive, lymphodepletion	T cell receptors or chimeric antigen receptor specific to mouse may not be specific to human therefore requires modification for clinical translation, antigens expressed are not always clinically relevant
Xenograft	Human tumors could be targeted	Impact of lymphodepletion, impact of endogenous immune system could not be studied, may not predict toxicity
Humanized	Recapitulate the best clinical scenario, readily translate to clinic	Expensive, immune system still may not totally be like syngeneic model

### Regional Delivery of T-Cell-Based Immunotherapy for CNS Tumors

**Figure 1:**
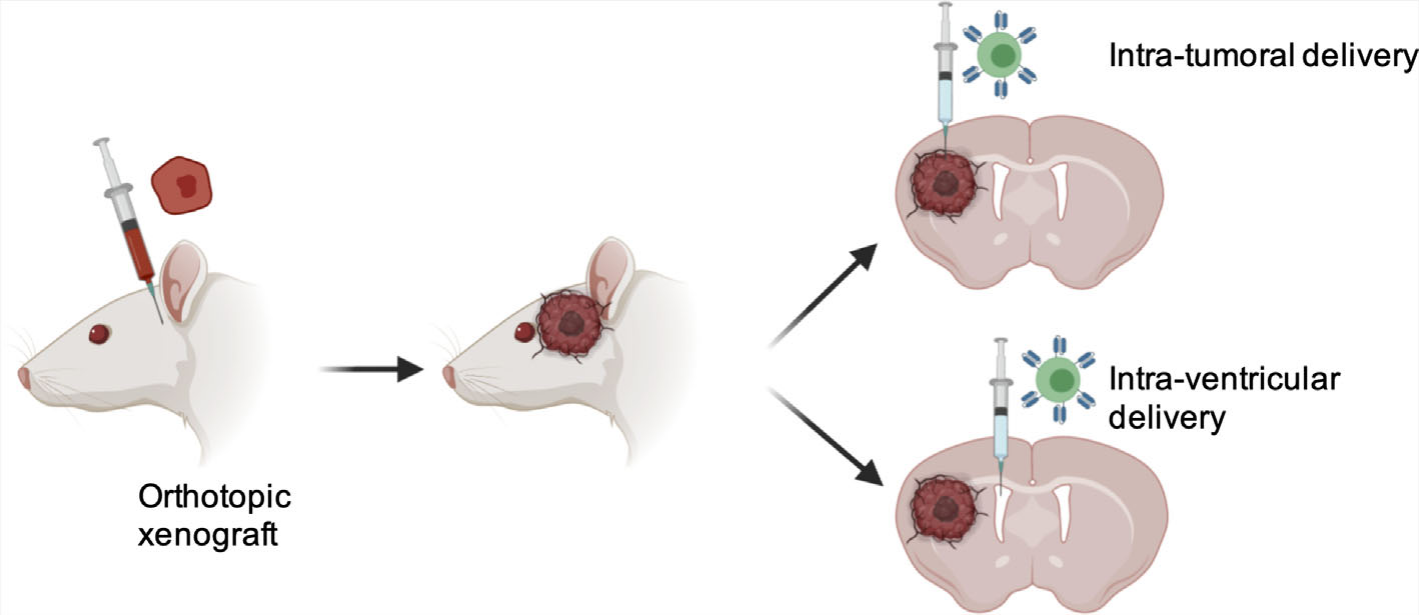
Advantages with locoregional delivery of T cells therapy into CNS tumors. 1) Overcoming BBB, 2) Increased tumor penetration; 3) Reduced number of T cells required; 4) Minimized chance of systemic toxicity.

Success of T cell-based therapy depends on the infiltration and effector functions of T cells at the tumor site. Like many other solid tumors, CNS tumors possess major barriers to infiltrating T cells. One approach to overcome this issue is the locoregional administration of T cells directly to the tumor site. Direct intratumoral delivery of engineered T cells is being tested in other solid cancers (58). In breast cancer, intratumoral injections were well tolerated and elicited inflammatory responses within tumors (59). A side-by-side comparison showed that intrapleurally administered mesothelin-targeted T cells outperformed systemically infused T cells in preclinical models (60). In CNS tumors, one patient showed regression of all intracranial and spinal tumors when multiple doses of IL13Rα2-targeted CART cells were administered directly into the tumor cavity and into the ventricular system; no increased toxicity was noted (50). In a preclinical glioma model, intracerebral delivery with a human, epidermal growth factor receptor variant III (EGFRvIII)-specific CAR T showed anti-glioma activity (61). In pediatric atypical teratoid/rhabdoid CNS tumor, Mackall’s group showed that B7-H3 expression is dramatically reduced in the postnatal brain and nonmalignant brain compared to the prenatal brain and malignant tumor. Using a preclinical mouse model, the most efficacious route of administration for B7-H3 CAR T was determined. Compared to the intravenous route, B7-H3 CAR T administered intratumorally and intracerebroventricularly showed multiple advantages. i) First, survival benefit was significantly higher; ii) Second, lower numbers of CART cells were required to cure the mice; iii) Third, Inflammatory cytokines such as IFN-γ, interleukin-4 (IL-4), IL-10 were not elevated, and; iv) Fourth, tumor penetration was significantly higher (62). Along the same line, Taylor and colleagues demonstrated in group 3 Medulloblastoma that intraventricular administration of EPHA2-targeting CAR T cells showed superior therapeutic effects, allowed penetration to the tumor and CAR T cell persistence due to continuous activation in the tumor site (63). Vora et al. administered CAR T cells against primary glioblastoma cell lines directly into the brain of tumor-bearing mice. CART133 cells showed improved efficacy in patient derived GBM xenograft models. Interestingly, CART133 showed superior efficacy in killing CD133+glioblastoma than antibody alone and dual antigen engager (DATE), indicating injecting adoptive T cell- derived therapy may serve better than antibody-based therapies that modulate T cells *in vivo* for treating GBMs (57). A major limitation of CART cells is that they use a single-chain variable fragment (ScFv) for antigen recognition. This is limited to only cell surface antigen. TCR-based immunotherapies are being developed to target intracellular proteins. It was shown that direct injection of TCR-transduced HLA-A2+ T cells efficiently regressed the progression of glioma xenografts in mice (51). As compared to other solid cancers, tumors of the CNS are subject to a specific degree of physical confinement and based on clinical and preclinical data, it is suggested that T cell-based therapy may be delivered locoregionally for CNS tumors (**Figure 1**).

Taken together, all these data showed that regardless of targets antigen, locoregional delivery of T cells to CNS tumors not only limits the toxicity due to systemic exposure but may also show enhanced therapeutic outcomes and warrants future clinical trials to consider regional delivery of T cell therapy to CNS tumors (**Table 2**).

**Table 2:** Preclinical and clinical utilization of locoregional delivery of T-cell-based therapy in non-GBM and GBM.

Therapy	Preclinical/clinical	Targeted Tumor	Remarks	References
**panErbB-specific CAR T cells**	Phase I clinical trial	Head and Neck Cancer	Safe intra-tumoral administration of T4 in patients with advanced HNSCC.	NCT01818323
**c-Met-CAR T**	Phase 0 clinical trial	Metastatic breast cancer	Intratumoral injections of mRNA c-Met-CAR T cells are well tolerated	NCT01837602
**M28z T**	Preclinical	Pleural malignancies	Intrapleurally administered M28z T cells eradicate pleural tumor	(60)
**IL13Rα2-CAR T**	Phase I clinical trial	Recurrent or Refractory Malignant Glioma	Intracavity and Intraventricular Well tolerated and One patient showed clinical response	NCT02208362 (50)
**EGFRvIII CART**	Preclinical	Glioblastoma	Intracerebral delivery, shows efficacious against human glioma	(61)
**B7-H3.BB.z-CAR T**	Preclinical	Atypical teratoid/rhabdoid tumors (ATRTs)	B7-H3.BB.z-chimeric antigen receptor (CAR) T cells injected intracerebroventricularly or intratumorally showed potent antitumor effects against cerebral ATRT xenografts in mice	(62)
**EPHA2 monovalent CAR T TRI CAR T (EPHA2, HER2 and IL-13Ra2)**	Preclinical	Recurrent medulloblastoma and ependymoma	Lateral ventricle administration of CART outperforms intravenous delivery	(63)
**CD133 CART**	Preclinical	Glioblastoma	CD133 CART showed enhanced efficacy against orthotopic GBM models	(57)

## To Improve the Persistence of T Cells in Immunosuppressive Microenvironment

As compared to blood cancers, the anticancer potential of transferred T cells is limited by its proliferative capacity at tumor sites in solid tumors such as CNS tumors. Importantly in the brain, to mitigate the effect of increased intracranial pressure and inflammatory response, the CNS utilizes immunosuppressive mechanisms at both cellular and molecular levels (64). As CNS tumors arise from the pre-existing immunosuppressive environment, development of T cell-based therapy that can persist at tumor sites and provide antitumor potential without elevating intracranial pressure and severe inflammatory reactions poses an additional challenge. To improve the persistent of T cells in CNS tumors, several approaches could be considered such as finding the best costimulatory molecules, incorporation of cytokines during T cell engineering, selection of T cells subsets that can persist longer in CNS tumors, designing T cells to utilize immunosuppressive molecules in favor of its proliferation, disruption of checkpoint molecules and preparing T cells that with greater metabolic fitness to survive in CNS tumor microenvironment.

Costimulatory molecules play a critical role in T cell proliferation. For example, first generation CART cells could not persist *in vivo* due to the lack of co-stimulatory domains. Later, costimulatory domains were incorporated into second-generation CAR T cells, which showed clinical efficacy and persistence (65). CART cells currently in clinical use for treating B cell malignancies include second generation CART with CD28 and 41BB costimulatory domains. It is becoming apparent that persistence of CART correlates with clinical efficacy and that CD28 costimulatory signaling supports robust effector function whereas 41BB supports memory function and long-term persistence (66–69). In non-small cell lung tumors, it was shown that encoding different costimulatory domains (for example, ICOS for CD4CART) showed enhanced persistence of CART cells in preclinical models. CD4CART containing an ICOS costimulatory domain enhanced persistence *in vivo*, which is supported by the persistence of CD8CART containing CD28 and 41BB domains (70). Third generation CAR incorporates two costimulatory domains. In the clinical trial for B cell Non-Hodgkin’s Lymphomas, third generation CAR i.e. incorporation of 41BB in addition to CD28 is associated with greater expansion and persistence. Interestingly, patients who are high risk of relapse with no measurable diseases seemed to benefit most (71). It would be interesting to evaluate whether singular costimulatory domain or combination of costimulatory domain in CAR will mediate survival benefits for CNS tumors.

Cytokines such as IL-15 play an important role in T cells activation, proliferation and cytokine secretion (72). Utilizing T cells to secrete cytokines directly to the tumor site may improve the persistence of T cells. Recent data suggested that IL-15 secretable CART and IL13Rα2-CAR.IL15 demonstrated survival advantages in U373 glioma orthotopic xenograft models compared to IL13Rα2-CAR. The overall survival advantage was achieved due to superior persistence, cytokine production and proliferative capacity of IL-15 secreting IL13Rα2-CAR.IL15 cells (73). In addition to cytokine IL-15, other cytokines such as IL-18, IL-12, IL-21, IL-36_γ_ could be evaluated to test the persistence of cytokine-armored T cells in the context of CNS tumors. As well, composition of T cells subsets impacts the therapeutic outcome of CNS tumors. Recent findings demonstrate that CD4+ and CD8+ CART cells behave differently against GBM. IL13Rα2- CD4+ CAR T outperformed IL13Rα2-CD8+ CART in GBM preclinical models. CD4+CART cells persisted longer and mediated direct cytotoxicity, suggesting that each subset of T cells has different persistence abilities (74). Many other strategies have been applied to enhance the persistence of CART cells in solid tumors.

In the tumor *milieu* of solid cancers, elevated levels of immune-suppressive cytokines and immune-inhibitory molecules are present. To convert immunosuppressive cytokines to stimulate T cells, CAR was designed to co-express an IL-4 extracellular domain and fused with an activating IL-7 intracellular domain. PSCA.4/7-ICR-CAR T and MUC1.4/7-ICR-CAR T were constructed and tested in pancreatic and breast tumor models (74, 75). This enhanced expansion and antitumor activity *in vitro* and *in vivo* showed that the immunosuppressive environment could be turned in favor of antitumor immunity by modifying CART design. Similar approaches could also be applied for other immunosuppressive cytokines such as TGF-beta (76).

Due to advancements in gene editing techniques such as CRISPR-Cas9 technology, it has now become possible to disrupt the gene of interest in transferred T cells. One such immune-inhibitory molecule of interest is PD1. During the manufacturing of CART cells, T cells must undergo an activation process, which leads to enhanced expression of PD1 on CART cells. PD1 binds to PDL1 or PDL2, which are expressed on the surface of tumor cells and prevents transmission of activating signals on T cells (77). A number of clinical trials are ongoing to disrupt PD1 in T cells in treatment of both blood and solid cancers (NCT 03747965, NCT03298828) (78). In addition to PD1, there may be several other immune checkpoint molecules such as TIGIT, CTLA-4, VISTA, TIM-3, LAG-3 expressed in T cells at baseline levels and during the process of activation, these checkpoint molecules are further upregulated. However, further studies are warranted to explore whether deleting any of these molecules from CART cells will add advantage to enhance the efficacy of transferred T cells against CNS tumors.

**Figure 2:**
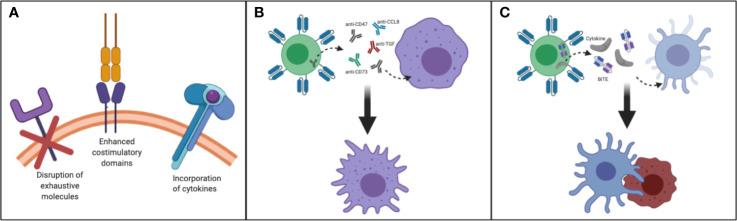
Making T cells persistent in TME of CNS tumors: **(A)** Disruption of exhaustive molecules, incorporation of cytokines and enhanced costimulatory domain **(B)** By designing T cells that secrete/express molecules such as anti-CD47, SIRPα, anti-CD73, anti-CCL8, anti-TGF/TGF-receptor, and FTL3 could be delivered to remodel myeloid rich CNS tumors such as GBM. This strategy may help to convert immunosuppressive TAM to immunostimulatory TAM. **(C)** By designing and manufacturing armored T cells capable of secreting growth factors needed to reactivate and redirect endogenous immune cells CNS tumors could be better targeted.

Taken together, to improve the persistence of transferred T cells for enhancing antitumor activity for patients with CNS tumors, factors such as incorporation of cytokines in CART construct, harnessing T cell subsets, enhanced costimulatory domains, reversing the immunosuppressive cytokine environment and disruption of exhaustive molecules should be taken into consideration (**Figure 2A**).

### Remodeling Myeloid Cell Environments

Myeloid cells, such as Tumor- associated macrophages (TAMs) play a dominant role in the brain tumor microenvironment (TME). It is becoming clear that CNS tumors such as GBM are myeloid- rich tumors. Chemokines such as CCL2 and cytokine colony-stimulating factor (CSF1) recruit circulating monocytes to the brain tumor microenvironment. Depending upon the TME, TAMs are either transformed to inflammatory TAMs which suppress tumor growth by producing cytokines such as IL-12 and TNF or become anti-inflammatory to support tumor growth by producing TGF-beta, IL-10, and arginase (79). Even in inflammatory conditions, tumor cells and TAMs upregulate checkpoint molecules such as PDL1 and Galectin which bind to transferred T cells and thus inhibit its effector function (80). Previously, it was shown that macrophages inhibit CD8 T cells from reaching tumors cells and limit the efficacy of anti-PD1 therapy in human and murine tumors (81). CD73^hi^ macrophages were identified in GBM and targeting CD73 reprograms the myeloid environment to be favorable for anti-tumor immunity (38). CD73 contributes to tumor immune escape by converting immune-activating ATP into immunosuppressive adenosine. CD73hi macrophages potentially suppress infiltrating immune cells through adenosine production (82).

CCL8 secreted by TAMs increased progression of GBM and elevated the expression of GBM stem cell markers such as CD133 and SOX2. CD47, a “marker-of-self” protein, forms a signaling complex with SIRPα that enables the don’t-eat-me signal. This negative signal inhibits phagocytosis mediated by macrophage (83). Chemoradiation treatment of GBM when combined with anti-CD47, showed enhanced effect of phagocytosis mediated by macrophages (79) and showed improved survival of mice with patient-derived orthotopic xenografts compared to TMZ or radiation alone (80). CD47 blockade has been shown to promote tumor cell phagocytosis and promising preclinical data for CNS tumors and clinical data for NHL (84, 85). In addition, anti-CD47 blockade also showed response in absence of infiltrating macrophage suggesting microglia are also effectors cells of CD47-SIRPa axis (86). Irradiation and TMZ also significantly enhanced anti-CD47 mediated phagocytosis of GBM, suggesting the benefits of combination therapy (87). For solid tumors such as brain tumors, one advantage of using T cells therapy is the feasibility of local delivery into tumor sites. In this perspective, it would be interesting to evaluate whether T cells secreting different molecules such as CD73, anti-CCL8, anti-CD47, SIRPα, anti-TGF/TGF-receptor reprogram TAMs and increase therapeutic benefit (88). Reprogramming the suppressive myeloid environment towards the proinflammatory state and harnessing the presence of TAMs rather than eliminating them from the tumor may improve the phagocytosis of myeloid cells and may also unleash endogenous T cell effector functions.

Overall, encouraging data indicates that modulating myeloid cells in CNS tumors such as GBM may improve the therapeutic outcome of immunotherapies. Strategies to reverse immunosuppressive macrophages into proinflammatory M1-like macrophages could have important implications for myeloid-rich tumors, and T cells could be utilized to deliver such agents to tip the balance towards the immunostimulatory M1 macrophage state (**Figure 2B**).

## Window of Treatment Opportunity: Directing T-Cell Therapy Toward Minimal Residual Disease in CNS Tumors

Standard of Care (SOC) treatment for CNS tumors such as GBM includes surgery followed by chemoradiotherapy. Despite this, recurrence occurs locally or distally. It has been shown that GBM cancer stem cells are resistant to chemoradiation and these cells are mainly responsible for recurrence (89, 90). After SOC, when the bulk of tumor is destroyed and before recurrence, when chemoresistant tumor cells have not fully grown into aggressive and unmanageable tumors, may represent the critical time to intervene with T cell-based personalized therapy. This stage is referred to as Minimal residual disease (MRD) (91).

Resistant cells that arise after chemoradiation may be expressing unique biomarkers for each patient. In GBM, intratumoral heterogeneity is very high and finding biomarkers unique to each patient may be challenging. However, liquid biopsy has gained increased attention in the cancer detection field due to a revolution in cell sorting technology and next generation sequencing platforms. The isolation of circulating tumor cells, circulating tumor DNA and exosomes has wide application for cancer diagnosis, screening and for detection of resistance to given treatments (92). Moreover, liquid biopsy is a non-invasive technique that offers advantages over repeated surgery for biopsy, which is not always possible for CNS tumors (93). Blood and cerebrospinal fluid (CSF) can be used for liquid biopsy samples in GBM (94). It was shown that liquid biopsy can detect underlying mutations GBM patients in genes such as *IDH1*, *EGFR*, *TP53*, *MET*, *PIK3CA*, *NOTCH1*, and *PTEN* (95, 96). CSF is considered safe to obtain and frequently accessed for certain brain cancers such as CNS lymphoma, MB and germ cell tumors for staging of tumors. Lumbar puncture is utilized to collect CSF, and this presents opportunities for diagnosis in patients with inoperable tumors, and also to study the continuous evolution of tumors throughout treatment. Bettegowda et al. reported a close representation of tumor-DNA when glioma samples were analyzed using CSF and neurosurgical tumor resection within a few weeks (95). Tumor-derived DNA was detected in half of the patients in CSF samples and was correlated with disease burden. They noticed co-deletion of chromosome arms 1p and 19q (1p/19q codeletion). Mutations in IDH genes were shared in all matched ctDNA-positive CSF/tumor pairs. Interestingly, evolution in growth factor receptor signaling pathways was observed. Previous studies have demonstrated the utility of CSF in identifying GBM biomarkers such as tenascin, osteopontin, nerve growth factor, IDH1/2, EGFR, PTEN, FGFR2 and ERBB2 (94, 97). There is mounting evidence to show that targeting MRD increases the cure rate in solid and hematological cancers (98, 99). It is expected that this noninvasive technology could be utilized to detect chemo-resistance biomarkers during the MRD stage. It was shown that persistence of disease is not due to the mutation, but due to drug treatments that enrich therapy-resistant tumor cells (99, 100). Identifying biomarkers and designing TCR/CART therapy and redirecting T cell-based therapy to such therapy- resistant cells may eliminate tumors and prevent recurrence. For example, CD133 has been used as a GBM stem cell marker (90, 100) and CD133+ GBM cancer stem cells were shown to be resistant to chemotherapy and radiotherapy (101). Vora et al. showed the efficacy of CD133CART in human tumors in orthotopic GBM xenografts, suggesting a possibility of targeting chemoradioresistant CD133+ cells specifically at MRD stage.

T cell-based therapies that treat patients during their MRD stage may have multiple advantages. First, there is a less suppressive TME present to inhibit transferred T cell function. Second, there are likely a smaller number of treatment refractory subclones present at the MRD stage such that low dose therapy may be sufficient to attack resistant tumor cells as compared to a fully-grown relapsed tumor. Third, chemotherapy such as Temozolomide induces lymphopenia (102). In hematological malignancies, it was shown that host lymphodepletion with chemotherapy enhances CAR T-cell proliferation and persistence (103). Therefore, it is expected that when T cell-based therapies are given at the MRD stage, this may lead to better persistence of transferred T cells.

**Figure 3:**
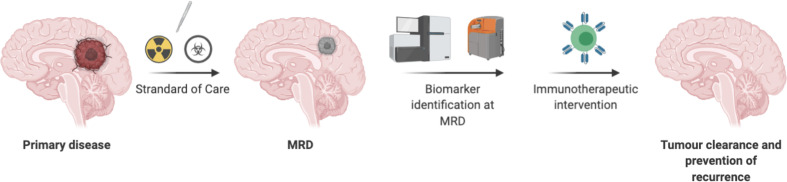
Directing T cells therapy toward minimal residual disease in CNS tumors have advantages due to less suppressive environment, may not need lymphodepletion, a smaller number of T cells may be required. However, chemo radio resistant tumor cells need to be identified as a biomarker to redirect T cells-based therapy.

In summary, identification of biomarkers using liquid biopsy may offer insights into therapy- resistant tumor cells for CNS tumors, and targeting these resistant tumor cells at MRD stage may lead to prevention of recurrence. Minimum target receptor density required to persist CART cells at MRD stage deserves further evaluation. Future considerations should be focused on treating MRD CNS tumors with T cell therapy (**Figure 3**).

## Harnessing The Endogenous Immune System

So far, the success of T cell therapy, such as CART, is limited only to certain hematological malignancies, and there is an urgent need to expand these therapies to solid cancers. Compared to hematological malignancies, expanding T cell- based therapies to CNS tumors is restricted due to challenges such as i) immunosuppressive microenvironment, ii) on- target off -tumor toxicity due to expression of antigens in normal organs such as brain, iii) antigen heterogeneity, and iv) BBB. Without involving endogenous immune system, it is unlikely that adoptive T-cell transfer will overcome all of these challenges and show efficacy in CNS tumors. Therefore, to enhance the success of adoptively transferred T cells for treating CNS tumors, tuning the endogenous immune system is critical. Previously, it was shown that presence of tumor infiltrating immune cells such as T cells, NK cells, and M1 macrophages were shown to increase the survival of patients (104–106). Along this line, Marcela Maus and colleagues demonstrated the enhanced efficacy of therapy when endogenous T cells were engaged by CART cells secreting BiTE (107). In this study, they reported that EGFRvIII CART secreting EGFR BiTE not only targeted EGFRvIII+positive GBM cells, but also wild type EGFR positive cells were targeted by untransduced bystander endogenous T cells through BiTE. This approach has advantages in targeting heterogeneous tumors. Paul Beavis and colleagues reported that adoptively transferred T cells expressing the DC growth factor Flt3L promoted the proliferation and differentiation of local dendritic cells and promoted endogenous antitumor T cell epitope spreading in solid cancers (108). This strategy may overcome tumor heterogeneity and should be given consideration to treat CNS tumors. Brown et al. showed that a GBM patient treated with IL13BBζ–CAR T cells showed presence of endogenous immune cells in cerebrospinal fluid. This study indicated the involvement of the endogenous immune system in IL13BBζ–CAR T-mediated antitumor responses. It was shown that endogenous immune cells and inflammatory cytokines were increased after each intraventricular infusion (50).

Taken together, augmenting the endogenous immune system could be a promising strategy to overcome the major clinical problems of CNS tumors such as antigen heterogeneity and the immunosuppressive environment. This could be achieved by developing armored T cells capable of secreting cytokines, growth factors and antibodies needed to activate anti-tumor endogenous immune cells (**Figure 2C**, **Table 3**).

**Table 3:** Secretion of immune-modulating molecules by CART cells.

Therapy	Secreted factors	Targeted tumor	Preclinical/clinical	Remarks	References
**Anti-CD47 VHH-FC secreted by anti-PDL-CART**	Anti-CD47 Fc	Melanoma	Preclinical	Delay of syngeneic tumor growth	(109)
**CART cells secreting BiTEs**	BiTEs: CD3-EGFR	Glioblastoma	Preclinical	Effective against heterogeneous tumors in mouse models of glioblastoma	(110)
**GD2CART**	IL-15	Neuroblastoma	Preclinical	Superior antitumor activity, enriched in stem cell like properties	(111)
**CART**	IL12	Metastatic colorectal cancer	Phase I/II clinical trial	Unpublished	NCT03542799
**Anti-CAIX CART**	Anti-PDL1	Renal cell carcinoma	Preclinical	Significant reduction of tumor growth	(112)
**19m28mz or 4H1m28mz**	PD-1 blocking scvf	PDL1+ hematologic and solid cancers	Preclinical	Similar or better efficacy when compared to CART+Inhibitor	(113)

## Designing Multivalent T*-*Cell Therapy for CNS Tumors

**Figure 4:**
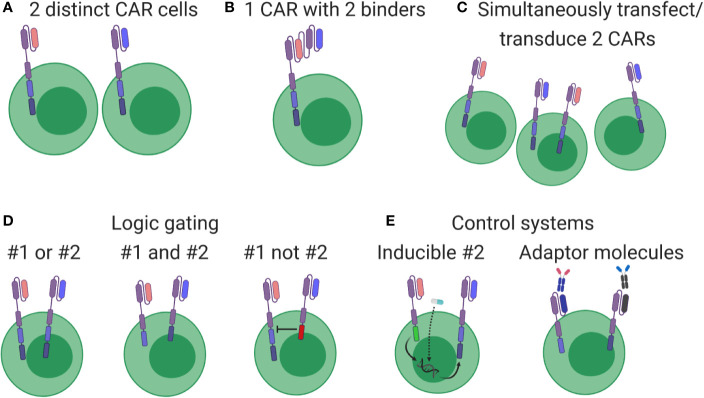
Overview of multi-targeting CAR formats. **(A)** Cells expressing different CARs (pink or blue) are produced separately and then pooled for patient administration. **(B)** Each cell expresses a CAR containing two binders, thereby driving cytotoxic responses when either antigen is detected. **(C)** Constructs are pooled before production, generating a cell product with mixed CAR expression. **(D)** Genuine multi-targeted approaches involving simple logic gates. Left: both CARs contain all signaling elements, leading to cytotoxic responses when either antigen is identified. Middle: signaling elements are split between CARs, leading to cytotoxic responses only when both antigens are present. Right: one CAR contains inhibitory signaling elements, thereby turning off the pink CAR when blue antigen is identified. **(E)** Multi-targeting strategies involving control systems. Left: both constructs are genome integrated, but transcription and expression of the blue CAR is induced by an exogenous drug (star) or through signaling events conducted through the pink CAR. Right: CARs without therapeutic antigen binders are integrated and expressed, but become functional only after administering an appropriate linking molecule.

Most CAR cell therapies currently under clinical investigation target a single antigen (i.e. CD19 *or* CD20 *or* HER2), with some exceptions (NCT03019055, NCT03448393, NCT03125577, NCT03287817, NCT03233854). However, simultaneously targeting multiple antigens was always considered a logical next step that could improve treatment efficacy by simply killing additional cancer cells or avoiding potential therapy-induced antigen loss (107). Multi-targeted CAR cell therapies take several forms, outlined in **Figure 4**. While multi-targeting CAR clinical trials involving CD19 plus CD20/CD22 have been launched in leukemia/lymphoma (**Figures 4A, B**), these formats represent early strategies with some advantages [(A): ratio of blue to pink cells can be adjusted pre-infusion] and weaknesses [(A): requires double the resources; (B): targets are identified equally; (C): introducing multiple viruses or plasmids might not meet regulatory guidelines]. Due to these logistical and potential functional limitations, other multi-targeting strategies utilizing so-called logic gates (**Figure 4D**) and other control systems (**Figure 4E**) are under intense development (114). In fact, various combining approaches in **Figures 4D, E** should allow autonomous or user-defined control over CAR cell therapies. However, although many of these strategies show promise in pre-clinical settings, they require significant context-specific engineering that depends on complex biology, including the antigen’s cancer specificity, binder affinity, respective CAR signaling contributions, anatomical location of cell delivery, and more.

CAR-T therapies for GBM targeting several different antigens are currently undergoing assessment in clinical trials, including: EGFRvIII (NCT02844062, NCT02664363, NCT01454596, NCT02209376), HER2 (NCT02442297, NCT01109095, NCT03500991), PD-L1 (using a switch receptor: NCT02937844), and IL13Ra2 (NCT02208362, NCT00730613, NCT01082926). CAR-Ts directed against EGFR (NCT03638167), HER2, (NCT03500991), and B7H3 (NCT04185038) are being tested in various brain tumors including gliomas and MB, and anti-GD2 CAR-Ts are undergoing assessment in DIPG (NCT04196413) and high-grade glioma (NCT04099797). In addition to this, several other targets have demonstrated promise in pre-clinical and clinical settings, such as CD133 (57), chlorotoxin (a cancer-binding peptide) (49), GD2 (115), and EphA2 (116). CAR T cells targeting Chlorotoxin are being evaluated in clinical trials in GBM (NCT04214392). Therefore, there appear to be ample candidate antigens in brain tumors against which multi-target CAR-T therapies can be designed. After assessing cell-specific expression of HER2, IL13Ra2, and EphA2 in patient biopsies, HER2- and IL13Ra2-targeting CAR-T cells showed increased ability to kill patient-matched cancer cells *in vitro* and eliminate U373 tumors in SCID mice (117). Furthermore, multi-CAR cells (**Figure 4D**) controlled tumor growth better than combining individual CAR-Ts (**Figure 4A**). When CARs were subsequently expressed against all three antigens simultaneously, these CAR-Ts showed increased responsiveness *in vitro* to patient-matched cancer cells and better controlled PDX growth in mice (118), highlighting their potential use in Group 3 MB and PFA ependymoma (63).

Despite these encouraging results, targeting multiple antigens is easier said than done. Notably, neurological toxicities have been observed in non-brain cancer cell therapy trials (119, 120), and extra caution must be considered when designing even single-target therapies for brain cancers. Cancers such as MB (121, 122) and GBM (123, 124) display dramatic molecular and genetic heterogeneity. While subgroup classifications might remain largely unchanged after recurrence for MB, SoC therapy significantly alters cellular evolution in GBM (125), further complicating potential antigen identification and target selection. In fact, while HER2/IL13Ra2/EphA2 triCAR-T cells show increased therapeutic efficacy in pre-clinical situations (63, 117), the actual expression of these markers across and between tumors and patients is relatively limited (126, 127), meaning their use in humans is likely restricted to specific patient populations. Expression of these and other markers in our (128) and others’ (49) primary patient tumor cell banks is highly diverse, making designing multi-targeted therapeutics that cover even these small sample sets seemingly difficult. To potentially bypass this problem, we are pursuing a strategy of targeting cancer stem cells in the hope their elimination restricts tumor cell self-renewal (57).

While selecting combinations of known brain tumor markers will likely result in efficacious therapeutics for specific patients, this is a relatively inefficient strategy. Instead, an attempt to rationally design single- and multi-CAR strategies by predicting safety profiles was recently published based on Human Protein Atlas (HPA) protein and RNA, Genotype-Tissue Expression (GTEx) RNA, and the database of differentially expressed proteins in human cancer (dbDEPC) (129). By looking for targets that are differentially expressed in healthy tissues, they identified gene pairs where at least one is a current clinical CAR candidate, thereby representing possible safe multi-CAR approaches (**Figure 4D**, middle panel). By performing the opposite comparison, they also identified gene pairs representing the right panel of **Figure 4D**, a strategy thought to best restrict on-target, off-tumor toxicity. This data is publicly available, so it is useful resource for researchers to easily find candidate targets that complement their current CAR of interest (129).

## Combinatorial Approaches to Car T-Cell-Based Therapies

One of the prominent challenges in treatment of brain tumors with CAR T cells is improving T cell persistence within the TME. Enhancing CAR T cell efficacy through blocking immune inhibitory molecules such as PD-1, PD-1L, and CTLA-4 has been investigated extensively in both preclinical and clinical settings (130). Several preclinical studies using glioma mouse models have demonstrated efficacy of immune checkpoint inhibitors (ICIs) in improving T cell infiltration and efficacy (131, 132). The early evidence from preclinical models was further substantiated by increased survival in glioma patients treated with neoadjuvant anti-PD-1 therapy (133, 134). The synergistic effects of ICIs and CAR T cells are currently being investigated in the clinical trials for GBM (NCT03726515; NCT04003649).

The SOC for patients with CNS malignancies has remained largely unchanged in the past decades and includes surgery, craniospinal irradiation and varying combinations of cytotoxic chemotherapies. Over the past years however, research has started to investigate the effects of chemoradiotherapies on the TME and its significance when incorporating immune cell-based therapies into treatment regimen. The first evidence of potential synergistic effects between stereotactic radio surgery and CAR T therapies came from a retrospective meta-analysis of patients with brain metastasis who were treated either with concurrent or sequential combination of ICS and SRS (135, 136). Patients in the concurrent cohort demonstrated an improved overall survival, although the mechanism by which radiation stimulates immunologic response remain under investigation. Several preclinical studies have demonstrated an increased tumor antigen expression post radiotherapy which in turns activated antigen presenting cells and subsequent activation of CD8+ cytotoxic T cells (137–140). Furthermore, radiation doses above 18Gy were shown to increase immunogenicity of cells through induction of Trex1 DNA exonuclease resulting in accumulation of cytosolic dsDNA and activation of cGAS/STING/INFβ pathway which in turn drives activation of CD8+ T cell-mediated response through recruitment of Batf3+ DCs. Combining a STING agonist with CAR T cells yielded an improved tumor response in preclinical models of pancreatic adenocarcinoma and warrants further preclinical investigation in the setting of CNS tumors (141).

The effects of the chemotherapeutic agents commonly used for treatment and management of CNS malignancies on CAR T cell activity remain to be fully elucidated. A study by Suryadevara et al. in 2018 demonstrated the enhanced activity, persistence and expansion of EGFRvIII CAR T cells post-TMZ driven lymphodepletion in syngeneic mouse models of GBM (142). Since TMZ remains the most common agent used in treatment of GBM, its lymphodepleting properties in combination with CAR T therapies need to be further assessed in the clinical trial setting.

**Figure 5:**
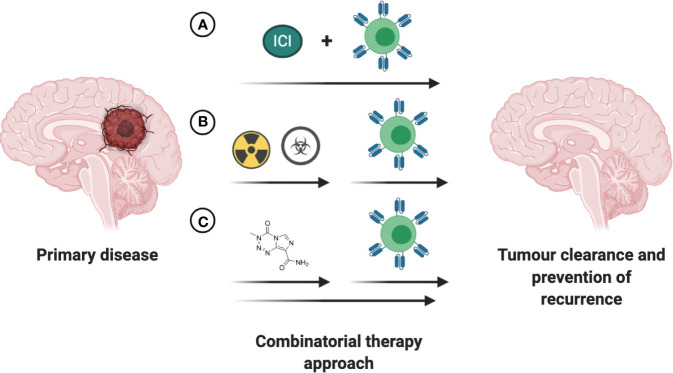
Increasing efficacy of immunotherapies through combination with existing FDA approved interventions. **(A)** Concurrent treatment with immune checkpoint inhibitors (ICI) holds promise to increase the anti-tumor efficacy of the immunotherapeutic intervention. **(B)** The time of immunotherapeutic intervention post completion of chemoradiotherapy has to be empirically determined based on antigen presentation, tumor burden and patient’s health status. **(C)** Small-molecule based treatments with intrinsic anti-tumoral effects or ability to enhance the anti-tumoral effects of immunotherapeutic may lead to greater reduction in tumor burden and prolong survival in patients with CNS tumors.

Over the past decade several small molecule-based therapies have been successfully translated into the clinic for treatment of aggressive brain tumors, including recurrent GBM. Although the combinatorial effects of small molecules with cell-based therapies are largely unknown and remain to be investigated in both preclinical and clinical trial settings, a few hold promise for positive synergy. One putative combinatorial treatment is Bevacizumab, an FDA-approved therapy for the treatment of recurrent GBM. As a disruptor of VEGF/VEGFR2 signaling, bevacizumab can potentially enhance CAR T cell therapy by reducing immunosuppressive effects of VEGF and promoting TIL trafficking (143, 144). Small molecule-based epigenetic modulators including HDAC and DMNT inhibitors have captured the interest of the research community in the past years, with the potential to overcome current immunotherapy limitations. The mechanistic insights and therapeutic implications of combining epigenetic modulators with immunotherapy in solid tumors has been extensively reviewed, amongst others, in Topper et al. and Aspeslagh et al. (145, 146). Despite the recent advances in our understanding of the intricate interplay between the established therapies for CNS tumors and CAR T based treatment (**Figure 5**), further clinical trial-based studies are urgently needed.

## Concluding Remarks

So far there is limited success in curing CNS tumors with T cell therapy. However, with the current advancement in knowledge of brain tumor biology and a parallel advance in techniques of engineering T cells, these therapies hold considerable promise in patients with CNS tumors. The CNS is a sensitive region for considering T cell-based therapies due to the possibility of T cells-based toxicities. Even though different preclinical models have their own limitations and there is no perfect model currently to test the toxicity of T cell therapy, there is an urgent need to find an innovative approach to test the toxicities of these therapies for CNS tumors. Importantly, sufficient consideration should be given to understand why T cell therapies given to patients have not yet shown efficacy. Whether it is due to lack of access to the tumor site, delivery route, length of treatment, or a problem with selecting antigen for T cells therapy remains to be determined. In context of systemic delivery, engineered T cells must reach tumor sites. During this process, engineered T cells need to interact with endothelial cells to pass the blood-brain barrier. This interaction may facilitate engineered T cells to reach tumor sites. Many strategies such as expressing chemokine receptors CXCR2/CCR2b, CXCR3 and use of oncolytic virus to secrete chemokines RANTES, IL-15 to drive CART cells to tumor sites are being actively tested to drive CART cells to tumor sites in non-GBM tumors (147–149). It has not yet been determined whether locoregional delivery alone will promote T cell homing to the tumor site, or whether additional cell-extrinsic factors will be required. The endogenous immune system present in the CNS could be manipulated through design of T cells. Finally, combinations with checkpoint therapy, standard therapy, metabolites, or sequential therapy may improve the survival rate of patients. Although there remains a profound challenge in treating CNS tumors with T cell- based therapy, success in treating B cells malignancies with CART has given hope for continuous improvement of strategies for T cell- based therapy against this deadly disease.

## Author’s Note

The figures were created using BioRender.com.

## Author Contributions

DU conceptualized the manuscript. DU, DBa, and DBl wrote the manuscript. PV, CV, and SS edited the manuscript. All authors contributed to the article and approved the submitted version.

## Funding

SS thanks the support from the Terry Fox Research Institute.

## Conflict of Interest

PV, CV, and SS are shareholders of Empirica Therapeutics.

The remaining authors declare that the research was conducted in the absence of any commercial or financial relationships that could be construed as a potential conflict of interest.
